# Accurate Identification of Common Pathogenic *Nocardia* Species: Evaluation of a Multilocus Sequence Analysis Platform and Matrix-Assisted Laser Desorption Ionization-Time of Flight Mass Spectrometry

**DOI:** 10.1371/journal.pone.0147487

**Published:** 2016-01-25

**Authors:** Meng Xiao, Lu Pang, Sharon C-A. Chen, Xin Fan, Li Zhang, Hai-Xia Li, Xin Hou, Jing-Wei Cheng, Fanrong Kong, Yu-Pei Zhao, Ying-Chun Xu

**Affiliations:** 1 Department of Clinical Laboratory, Peking Union Medical College Hospital, Chinese Academy of Medical Sciences, Beijing, China; 2 Department of Clinical Laboratory, Peking University First Hospital, Beijing, China; 3 Centre for Infectious Diseases and Microbiology Laboratory Services, ICPMR–Pathology West, Westmead Hospital, University of Sydney, Westmead, New South Wales, Australia; 4 Department of General Surgery, Peking Union Medical College Hospital, Chinese Academy of Medical Sciences, Beijing, China; The University of Hong Kong, HONG KONG

## Abstract

Species identification of *Nocardia* is not straightforward due to rapidly evolving taxonomy, insufficient discriminatory power of conventional phenotypic methods and also of single gene locus analysis including 16S rRNA gene sequencing. Here we evaluated the ability of a 5-locus (16S rRNA, *gyrB*, *secA1*, *hsp65* and *rpoB*) multilocus sequence analysis (MLSA) approach as well as that of matrix-assisted laser desorption ionization-time of flight mass spectrometry (MALDI-TOF MS) in comparison with sequencing of the 5’-end 606 bp partial 16S rRNA gene to provide identification of 25 clinical isolates of *Nocardia*. The 5’-end 606 bp 16S rRNA gene sequencing successfully assigned 24 of 25 (96%) clinical isolates to species level, namely *Nocardia cyriacigeorgica* (n = 12, 48%), *N*. *farcinica* (n = 9, 36%), *N*. *abscessus* (n = 2, 8%) and *N*. *otitidiscaviarum* (n = 1, 4%). MLSA showed concordance with 16S rRNA gene sequencing results for the same 24 isolates. However, MLSA was able to identify the remaining isolate as *N*. *wallacei*, and clustered *N*. *cyriacigeorgica* into three subgroups. None of the clinical isolates were correctly identified to the species level by MALDI-TOF MS analysis using the manufacturer-provided database. A small “in-house” spectral database was established incorporating spectra of five clinical isolates representing the five species identified in this study. After complementation with the “in-house” database, of the remaining 20 isolates, 19 (95%) were correctly identified to species level (score ≥ 2.00) and one (an *N*. *abscessus* strain) to genus level (score ≥ 1.70 and < 2.00). In summary, MLSA showed superior discriminatory power compared with the 5’-end 606 bp partial 16S rRNA gene sequencing for species identification of *Nocardia*. MALDI-TOF MS can provide rapid and accurate identification but is reliant on a robust mass spectra database.

## Introduction

*Nocardia* species are ubiquitous environmental bacteria that cause suppurative infections in humans, including in the lung, central nervous system and skin [1]. Nocardiosis typically occurs in immunosuppressed patients such as organ and stem cell transplantation, and malignancy, but also affects immunocompetent hosts [2–4]. Since there are species-specific differences with regard to geography and disease patterns, identification of *Nocardia* to species level is important to determine both epidemiology and clinical associations.

Conventional culture identification of *Nocardia* based on phenotypic methods and distinct (but limited) antimicrobial susceptibility profiles lacks sufficient discriminatory power, is slow and time-consuming and requires staff expertise [5, 6]. Thus identification of *Nocardia* isolates by molecular methods is increasingly used, of which PCR-based methods combined with DNA sequencing are the most popular. Of these, 16S rRNA gene sequencing is considered the “gold-standard” [6, 7]. However, 16S rRNA gene sequencing is unable to distinguish certain closely related species due to insufficient interspecies gene polymorphisms [6, 8], whilst also unable to resolve certain species, e.g. *Nocardia nova* due to the presence of multiple yet different copies of this gene [9]. To overcome this limitation, other gene polymorphisms have been evaluated, such as those within the β-subunit of the type II DNA topoisomerase gene (*gyrB*) [10, 11], the subunit A of the SecA preprotein translocase gene (*secA1*) [12, 13], the 65-kDa heat shock protein gene (*hsp65*) [14, 15], and the β subunit of DNA-dependent RNA polymerase gene (*rpoB*) [11]. A multilocus sequence analysis (MLSA) scheme analyzing sequence polymorphisms of multiple *Nocardia* genes was reported to be accurate not only for the identification of known *Nocardia* species but the unveiling of novel species [16].

Other than genomic approaches, proteomic methods, most notably matrix-assisted laser desorption ionization-time of flight mass spectrometry (MALDI-TOF MS) method, have also been evaluated to identify *Nocardia* species in clinical laboratories. However, the presence of aliphatic acids in the cell wall of *Nocardia* has posed an obstacle in achieving satisfactory protein profiles. Furthermore, although improvements are continuing, many databases provided by commercial MALDI-TOF MS systems contain only a limited number of archived *Nocardia* spectral profiles [17, 18]. Previous studies have reported identification to species level in only 14.9–80.4% of isolates [18–23]. Complementation with profiles provided by “in-house” databases, which in turn relies on knowledge of local epidemiology, hence may assist with identification to the species, or even, genus level.

In the present study, we firstly evaluated a published MLSA scheme [16] employing polymorphisms in the 16S rRNA, *gyrB*, *secA1*, *hsp65* and *rpoB* loci for the identification of clinical *Nocardia* isolates in our laboratory; and secondly, determined the ability of MALDI-TOF MS for species assignment. Results were compared to those obtained by the 5’-end 606 bp 16S rRNA gene sequencing. An “in-house” database of *Nocardia* protein profiles was established and evaluated for its ability to complement a commercial database for identification of *Nocardia* species.

## Materials and Methods

### Ethics

The study was approved by the Human Research Ethics Committee of Peking Union Medical College Hospital (PUMCHBC-C-2-Q01-1). Written informed consent was obtained from patients for the use of the samples in research.

### *Nocardia* strains and reference sequences

Twenty-five clinical strains were studied in the evaluation of the ability of an MLSA scheme [16] and MALDI-TOF MS to provide species identification. Isolates were cultured from patients admitted to the Peking Union Medical College Hospital from January 2009 to January 2015 (Table 1). All isolates were identified by standard phenotypic methods [24]. Isolates were stored at -80°C and subcultured on Columbia blood agar for 72 h to 96 h at 37°C to ensure adequate growth before study.

**Table 1:** *Nocardia* isolates examined (n = 25) in the present study.

Strains no.	Age (years)	Gender	Medical department	Specimen type	Immuno-Compromised (Yes/No)
PUNC001	53	Male	Outpatient	PICC Drainage	No
PUNC002	70	Female	Gastroenterology	Sputum	No
PUNC003	53	Male	Immunology	Lung tissue	Yes
PUNC004	64	Male	Respiratory	Subcutaneous nodule	Yes
PUNC005	72	Male	Nephrology	Sputum	Yes
PUNC006	31	Female	Emergency	Sputum	Yes
PUNC007	43	Female	Thoracic Surgery	Sputum	No
PUNC008	50	Male	Emergency	Hydrothorax fluid	Yes
PUNC009	67	Male	Emergency	Sputum	No
PUNC010	70	Male	Respiratory	Sputum	Yes
PUNC011	70	Male	Respiratory	Sputum	No
PUNC012	51	Male	Respiratory	Sputum	No
PUNC013	76	Male	Respiratory	Sputum	No
PUNC014	45	Female	Respiratory	Lung tissue	No
PUNC015	61	Female	Immunology	Hydrothorax fluid	Yes
PUNC016	81	Male	Outpatient	Sputum	No
PUNC017	71	Male	Respiratory	Sputum	Yes
PUNC018	56	Female	Respiratory	BALF	Yes
PUNC019	53	Male	Orthopedics	Pus	No
PUNC020	46	Female	Outpatient	Pus	No
PUNC021	57	Male	Immunology	Sputum	Yes
PUNC022	32	Female	Respiratory	BALF	Yes
PUNC023	45	Male	Emergency	Sputum	No
PUNC024	37	Male	Emergency	Pus	No
PUNC025	65	Male	Emergency	Sputum	No

Abbreviations: PICC, peripherally inserted central catheter; BALF, bronchoalveolar lavage fluid; SLE, systemic lupus erythematosus; COPD, chronic obstructive pulmonary disease.

In addition, the GenBank sequences of 16S rRNA, *gyrB*, *secA1*, *hsp65* and *rpoB* loci corresponding to 20 *Nocardia* type strains were studied as the “validation cohort” to determine the ability of the MLSA typing scheme [7, 16] to identify *Nocardia* clinical isolates collected (see S1 Table).

### DNA extraction, PCR amplification and sequencing

DNA extraction of all isolates was performed as previously described [8]. The *Nocardia* 16S rRNA gene was amplified using the primer pair 27F and 1522R [25]. The *Nocardia gyrB*, *secA1*, *hsp65* and *rpoB* genes were amplified as previously described [16]. In all cases, amplified PCR products were sequenced in both directions using the amplification primers on the ABI 3730XL platform (Applied Biosystems, Foster City, CA).

### Species identification by the 5’-end 606 bp partial 16S rRNA gene sequencing and MLSA

Molecular-based species identification was initially carried out by analysis of the 5’-end 606 bp partial 16S rRNA gene sequences as described using a percentage similarity (or identity) score of ≥ 99% as the criterion to strictly classify an isolate to species level [7]. MLSA was then carried out examining nucleotide polymorphisms in the partial 16S rRNA gene at 5’-end (462 bp), as well as the *secA1* (445 bp), *gyrB* (482 bp), *rpoB* (400 bp) and *hsp65* (401 bp) genes [16]. The concatenated *gyrB*-16S-*secA1*-*hsp65*-*rpoB* sequences (2190 bp) were then used for phylogenetic analysis by the maximum-likelihood algorithm based on the Tamura-Nei model [26] with 1000 bootstrap replication to ensure robustness using MEGA software (version 6.0, MEGA Inc., Englewood, NJ). DnaSP software (version 5.1, University of Barcelona, Spain) was used to assess the haplotypes of each gene [27]. Determining of potential subtypes of *N*. *cyriacigeorgica* isolates was further carried out by analysis of the *Nocardia hsp65* gene as previously described [28, 29].

### Establishment and validation of an “in-house” *Nocardia* mass spectrum database for MALDI-TOF MS

All *Nocardia* isolates was pretreated using a mechanical disruption-ethanol extraction method as described by Dunne *et al*. [30]. MALDI-TOF MS analysis of 25 clinical *Nocardia strains* was first carried by the Bruker Autoflex Speed TOF/TOF MS system using Biotyper software version 3.1 (db 4613, Bruker Daltonics, Billerica, USA) according to the manufacturer’s instructions. A spectral score of < 1.70 was considered not to provide reliable identification. A score of ≥ 1.70 but < 2.00 indicated identification at the genus level, and a score of ≥ 2.00 was considered identification at the species level [18].

Because the identification results using the manufacturer-provided Biotyper database was unsatisfactory (see “Results” below), five of the 25 clinical isolates representing five different *Nocardia* species (as identified by DNA sequencing in the present study—strain ID no. PUNC001 (*Nocardia farcinica*), PUNC002 (*Nocardia abscessus*), PUNC006 (*Nocardia cyriacigeorgica*), PUNC020 (*Nocardia wallacei*), and PUNC024 (*Nocardia otitidiscaviarum*); Table 2) were used to generate an “in-house” mass spectrum fingerprint database following the methodology of Segawa *et al*. [18]. Each isolate was analyzed in replicates of eight (ie. inoculated in eight spots on the target plate), and each spot was interrogated three times. This procedure was repeated at least once on a different occasion under identical test conditions. The resulting data were averaged using the Biotyper software v3.1 (Bruker Daltonics), a single mass spectrum representing each isolate was generated, and the resultant profiles established as a small “in-house” database.

**Table 2:** Identification results of 25 *Nocardia* clinical isolates by DNA sequencing and MALDI-TOF MS after interrogation against the Biotyper database (version 3.1) and the “in-house” database.

Strain ID no.	Identification by MLSA	Identification by conventional methods	Identification by MALDI-TOF MS^a^	
			First reported species	Score
**Isolates used for establishment of “in-house” mass spectra database (no. = 5)**				
PUNC001^a^	*N*. *farcinica*	*Nocardia* sp.	N/A	N/A
PUNC002^a^	*N*. *abscessus*	*Nocardia* sp.	N/A	N/A
PUNC006^a^	*N*. *cyriacigeorgica*	*Nocardia* sp.	N/A	N/A
PUNC020^a^	*N*. *wallacei*	*Nocardia* sp.	N/A	N/A
PUNC024^a^	*N*. *otitidiscaviarum*	*N*. *otitidiscaviarum*	N/A	N/A
**Isolates used for validation of the MALDI-TOF MS system^a^ (no. = 20)**				
PUNC003	*N*. *cyriacigeorgica*	*N*. *cyriacigeorgica*	*N*. *cyriacigeorgica*	2.160
PUNC004	*N*. *farcinica*	*Nocardia* sp.	*N*. *farcinica*	2.332
PUNC005	*N*. *cyriacigeorgica*	*Nocardia* sp.	*N*. *cyriacigeorgica*	2.129
PUNC007	*N*. *cyriacigeorgica*	*N*. *asteroides*	*N*. *cyriacigeorgica*	2.393
PUNC008	*N*. *farcinica*	*Nocardia* sp.	*N*. *farcinica*	2.411
PUNC009	*N*. *farcinica*	*N*. *farcinica*	*N*. *farcinica*	2.284
PUNC010	*N*. *cyriacigeorgica*	*N*. *cyriacigeorgica*	*N*. *cyriacigeorgica*	2.011
PUNC011	*N*. *cyriacigeorgica*	*N*. *cyriacigeorgica*	*N*. *cyriacigeorgica*	2.232
PUNC012	*N*. *farcinica*	*Nocardia* sp.	*N*. *farcinica*	2.196
PUNC013	*N*. *cyriacigeorgica*	*N*. *cyriacigeorgica*	*N*. *cyriacigeorgica*	2.126
PUNC014	*N*. *farcinica*	*N*. *farcinica*	*N*. *farcinica*	2.124
PUNC015	*N*. *farcinica*	*N*. *farcinica*	*N*. *farcinica*	2.055
PUNC016	*N*. *cyriacigeorgica*	*N*. *cyriacigeorgica*	*N*. *cyriacigeorgica*	2.422
PUNC017	*N*. *abscessus*	*N*. *abscessus*	*N*. *abscessus*	1.894
PUNC018	*N*. *cyriacigeorgica*	*Nocardia* sp.	*N*. *cyriacigeorgica*	2.307
PUNC019	*N*. *cyriacigeorgica*	*N*. *cyriacigeorgica*	*N*. *cyriacigeorgica*	2.145
PUNC021	*N*. *cyriacigeorgica*	*Nocardia* sp.	*N*. *cyriacigeorgica*	2.079
PUNC022	*N*. *farcinica*	*Nocardia* sp.	*N*. *farcinica*	2.498
PUNC023	*N*. *farcinica*	*N*. *farcinica*	*N*. *farcinica*	2.431
PUNC025	*N*. *cyriacigeorgica*	*Nocardia* sp.	*N*. *cyriacigeorgica*	2.052

Abbreviation: N/A, not applicable.

^a^ Results by using the Bruker Biotyper version 3.1 (Bruker Daltonics) complementation with the “in-house” database (see Materials and Methods for detail).

The 20 remaining clinical isolates were then analyzed against the Biotyper version 3.1 database (Bruker Daltonics) complemented with the “in-house” database. The technicians performing the analysis were blinded to the DNA sequencing results to avoid potential bias.

A main spectrum profile (MSP) dendrogram was further constructed using spectra of 25 *Nocardia* clinical strains in this study along with 37 reference spectra of 32 *Nocardia* species already contained in the Biotyper version 3.1 database for analysis of genetic relatedness.

### Review for publications on validation of MALDI-TOF MS in identification of *Nocardia* species

Key words “*Nocardia*” and “matrix-assisted laser desorption ionization-time of flight mass spectrometry” were used to search related publications in English archived in NCBI PubMed database (http://www.ncbi.nlm.nih.gov/pubmed, till Dec 31^st^, 2015). All complete articles of the search (16 publications in all) were screened. After excluding case reports, six publications were reviewed in detail.

### GenBank accession numbers

The DNA sequences of the full-length 16S rRNA, *secA1*, *gyrB*, *rpoB* and *hsp65* genes of 25 *Nocardia* isolates studied have been submitted to GenBank database (accession numbers KT985911 to KT985935, KU052160 to KU052184, KU052085 to KU052109, KU052135 to KU052159 and KU052110 to KU052134 for the above five genes, respectively, S2 Table).

## Results

### Identification by the 5’-end 606 bp partial 16S rRNA gene sequencing

Of 25 *Nocardia* clinical strains, 24 isolates representing four *Nocardia* species were identified unambiguously by the 5’-end 606 bp partial 16S rRNA gene sequencing. *N*. *cyriacigeorgica* (n = 12, 48%) and *N*. *farcinica* (n = 9, 36%) were the most common species, followed by *N*. *abscessus* (n = 2, 8%) and *N*. *otitidiscaviarum* (n = 1, 4%). However, the 5’-end 606 bp partial 16S rRNA gene sequence of strain PUNC020 showed 99% sequence similarity to both *N*. *wallacei* type strain DSM 45136^T^ (606/606, 100%) and *Nocardia transvalensis* type strain NRRL B-16037^T^ (600/606, 99.0%), though was identical to the former.

### MLSA

**Fig 1:**
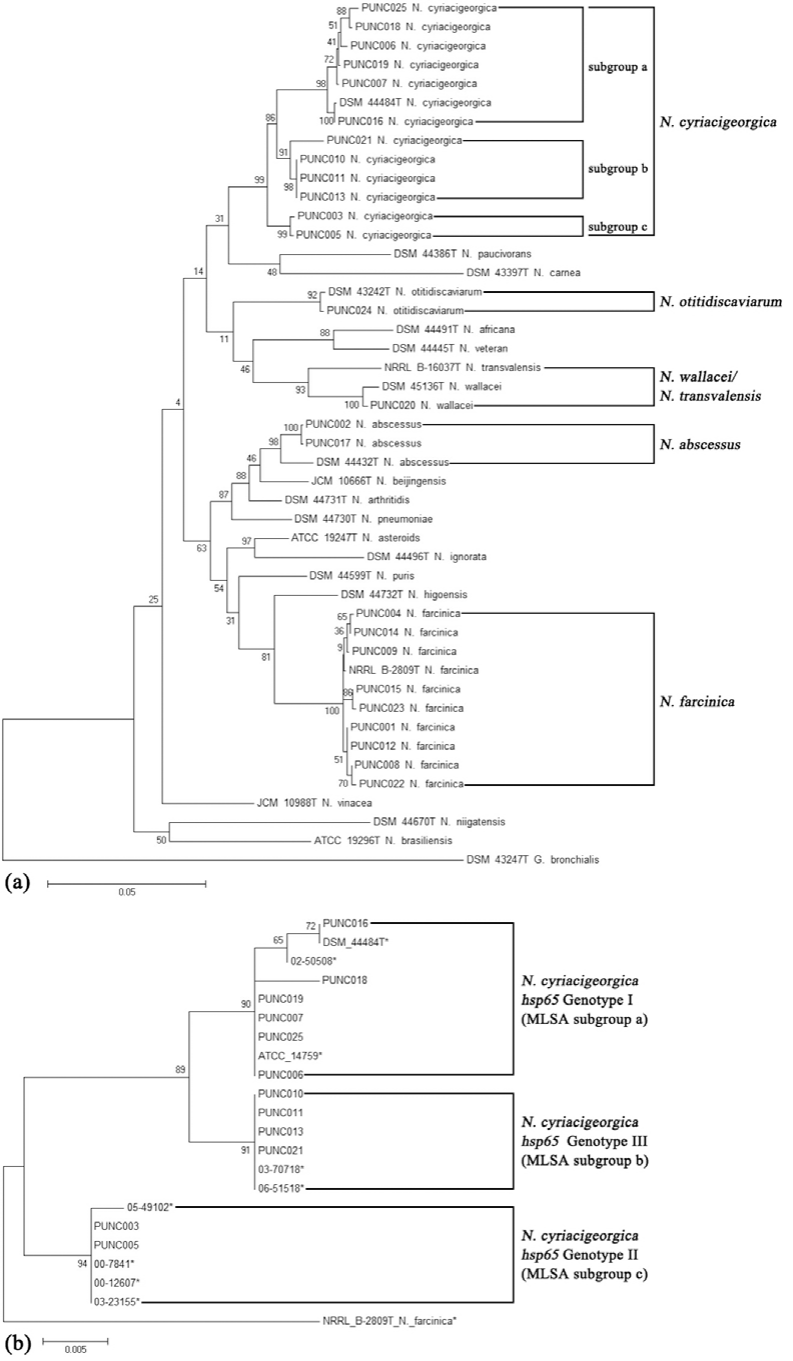
Phylogenetic trees are shown and were conducted using the maximum-likelihood method. **Fig 1a.** Phylogenetic tree based on the concatenated *gyrB*-16S-*secA1*-*hsp65*-*rpoB* sequences of 25 *Nocardia* clinical isolates and 20 *Nocardia* type strains, using *Gordonia bronchialis* strain ATCC 25592^T^ as an outgroup (S1 Table). **Fig 1b.** Phylogenetic tree based on the *hsp65* gene sequences of 12 *N*. *cyriacigeorgica* clinical isolates and nine *Nocardia cyriacigeorgica* isolates whose genotypes have previously been determined by Schlaberg *et al*. [29] using the sequence of a *Nocardia farcinica* strain as an outgroup.

Phylogenetic analysis of concatenated *gyrB*-16S-*secA1*-*hsp65*-*rpoB* sequences obtained from the 25 clinical isolates and 20 *Nocardia* type strain sequences clearly revealed five major clusters with bootstrap threshold values of ≥ 90% (Fig 1). Each cluster corresponded to a unique *Nocardia* species, ie. *N*. *cyriacigeorgica*, *N*. *otitidiscaviarum*, *N*. *farcinica*, and *N*. *abscessus*), except for clinical strain PUNC020 that was clustered together with type strains of *N*. *wallacei* and *N*. *transvalensis*. As strain PUNC020 was more phylogenetically closely related to *N*. *wallacei* strain DSM 45136^T^ compared with *N*. *transvalensis* strain NRRL B-16037^T^ (Fig 1), the isolate was assigned as *N*. *wallacei*.

Twelve *N*. *cyriacigeorgica* clinical isolates could further be classified into three subgroups: subgroup a (n = 6), b (n = 4) and c (n = 2), (Fig 1A). Comparing with results obtained by *N*. *cyriacigeorgica* genotyping using sequence analysis of the *hsp65* gene [28, 29], isolates belonging to *N*. *cyriacigeorgica* MLSA subgroups a, b and c corresponded to *N*. *cyriacigeorgica hsp65* genotype I, III and II, respectively (Fig 1B).

Overall, it was observed that the *rpoB*, *gyrB* and *secA1* genes were the most polymorphic (14 to 16 haplotypes identified amongst 25 clinical strains, haplotype diversity ≥ 0.950), followed by *hsp65* (10 haplotypes, haplotype diversity = 0.837), whilst the partial 16S rRNA gene was the least polymorphic (five haplotypes, haplotype diversity = 0.657) (Table 3 and S2 Table). Different degrees of intra-species micro-heterogeneity of different genes were also observed (Table 3). *N*. *cyriacigeorgica* isolates generally exhibited higher intra-species diversity than other species e.g. *N*. *farcinica* (Table 3).

**Table 3:** Genetic polymorphisms contained within the 16S rRNA, *gyrB*, *secA1*, *hsp65* and *rpoB* genes for 25 clinical *Nocardia* isolates studied.

Characters	*gyrB*	16S rRNA gene	*secA1*	*hsp65*	*rpoB*
**All clinical isolates (n = 25)**					
No. of haplotypes	16	5	14	10	16
Haplotype diversity	0.957	0.657	0.950	0.837	0.963
***N*. *cyriacigeorgica* (n = 12)**					
No. of haplotypes	8	1	6	5	8
Haplotype diversity	0. 924	N/A	0. 848	0. 803	0. 924
***N*. *farcinica* (n = 9)**					
No. of haplotypes	4	1	5	1	5
Haplotype diversity	0. 778	N/A	0. 889	N/A	0. 861

Abbreviation: N/A, not applicable.

### Improvement of MALDI-TOF MS identification capacity by the “in-house” database

Of the five species identified by the 5’-end 606 bp partial 16S rRNA gene sequencing and MLSA from the present study which we selected to establish a mini “in-house” database, representation of four species (*N*. *cyriacigeorgica*, *N*. *farcinica*, *N*. *abscessus* and *N*. *otitidiscaviarum*) was already included in the Biotyper version 3.1 database (Bruker Daltonics); however, *N*. *wallacei* was not represented. When all 25 clinical isolates were interrogated using only the Biotyper database, only eight of 25 (32%) isolates was identified to genus level, none (0%) to species level, and 17 isolates were not identified (68%).

Using the “in-house” database to complement the Biotyper database, 19 of 20 (95%) *Nocardia* clinical isolates were correctly identified to species level with scores of ≥ 2.0 (Table 2). *N*. *abscessus* strain PUNC017, which yielded a MALDI-TOF MS score of 1.894, was identified to genus level (Table 2).

**Fig 2:**
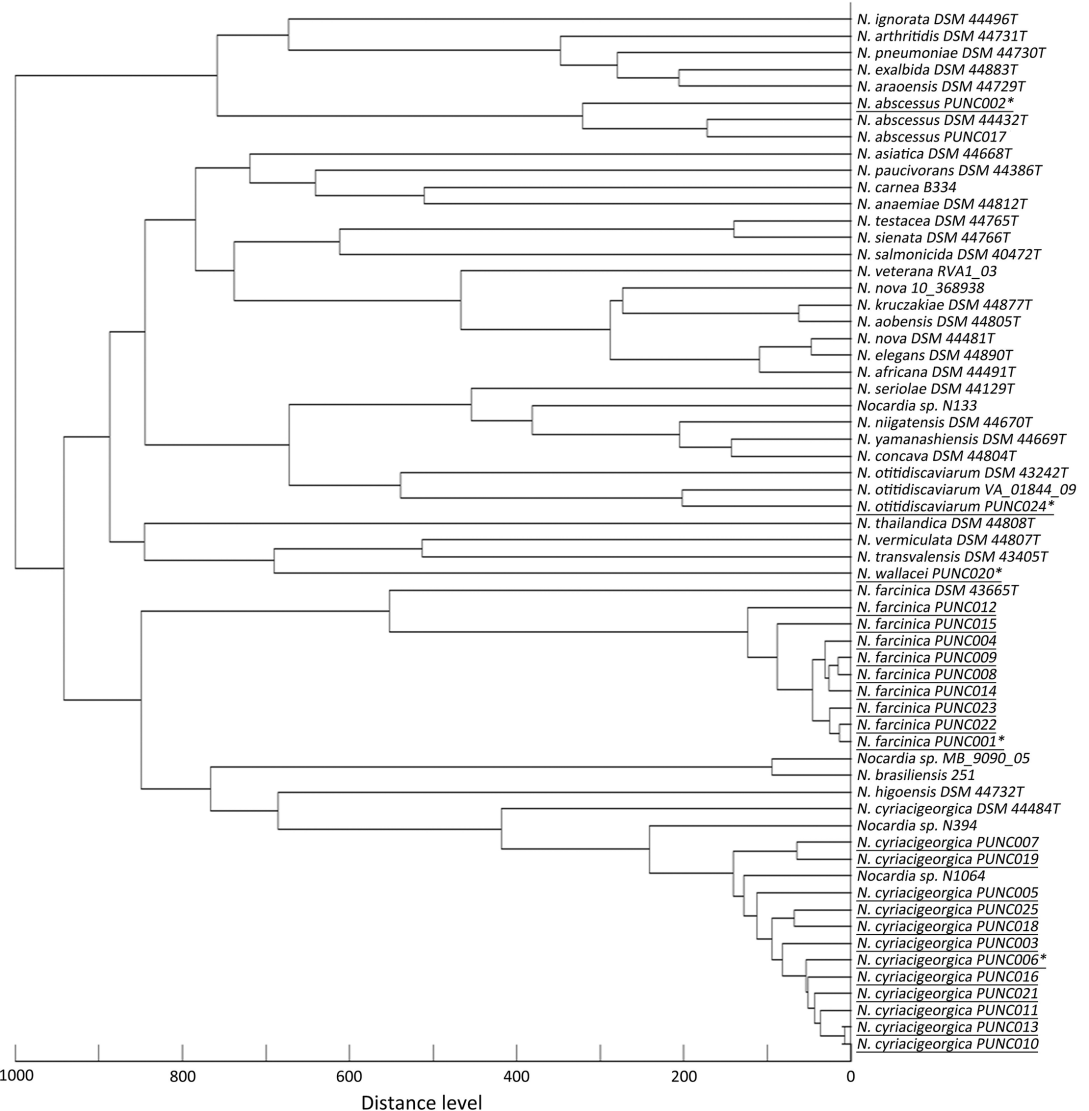
The main spectrum profile (MSP) dendrogram constructed using spectra of 25 *Nocardia* clinical strains along with 37 reference spectra of 32 *Nocardia* species contained in the original Biotyper database (version 3.1; Bruker). Strain identification numbers of clinical isolates collected in the present study are shown underlined, and isolates used for establishment of the “in-house” database was labeled with asterisks.

The MSP dendrogram indicated that the spectral profile of clinical isolates were distinct from the corresponding reference spectra for its species in the Biotyper database (Fig 2). The mass spectra differences between *N*. *abscessus* strain PUNC002 (for establishment of the “in-house” database) and *N*. *abscessus* strain PUNC017 (for test of the “in-house” database) also resulted in failure in identifying strain PUNC017 to specie level using the “in-house” database (Fig 2). Therefore, the mass spectra of strain PUNC017 was incorporated into the “in-house” database for future study. Similar divergence of mass spectra were also noted in *N*. *otitidiscaviarum* for clinical strain PUNC024 versus strain VA_01844_09 and type strain DSM 43242^T^ that included in the commercial database (Fig 2), which led to failure of identifying strain PUNC024 to species level using the commercial database.

### Literature review for *Nocardia* identification by MALDI-TOF MS

Of six studies reviewed, five had evaluated the Bruker Biotyper commercial database (version 3.1 or 3.0.2) and one, an Andromas (Paris, France) system (Table 4). The Biotyper database only correctly identified 14.9–52.8% of isolates to species level and an additional 6.9–52.7% of isolates to genus level. The performance of Andromas system (Paris, France) was better (80.4% to species level and 17.6% to genus level). Three studies further developed local “in-house” databases, and the correct identification rate was significantly improved to 79.1–90.6% to species level and an additional 4.7–11.5% to genus level (Table 4).

**Table 4:** Review of publications for evaluation of MALDI-TOF MS for the identification of *Nocardia* species.

				Commercial DB		"In-house" DB		
System	Reference method	ID criteria to species/genus	Ni/Ns for DB validation	DB version	Correct ID to species/genus	Ni/Ns for DB development	Correct ID to species/genus	Reference
Bruker Autoflex Speed	MLSA	≥ 2.0/< 2.0 and ≥ 1.7	See note^a^	BioTyper ver. 3.1	0%/32.0%^a^	5/5	95.0%/5.0%^a^	This study
Bruker microflex LT	16S rRNA gene and conventional	≥ 2.0/< 2.0 and ≥ 1.7	148/15	BioTyper ver. 3.1	41.9%/15.5%	232/53	89.9%/4.7%	[23]
Bruker (Model not specified)	16S rRNA, *hsp65* and *secA1* genes	≥ 1.9/< 1.9 and ≥ 1.7	87/25	BioTyper ver. 3.1	52.8%/6.9%	13/13	82.8%/11.5%	[22]
Bruker microflex LT	16S rRNA gene and conventional	≥ 2.0/< 2.0 and ≥ 1.7	64/22	BioTyper ver. 3.1	15.6%/25.0%	192/73	90.6%/9.4%	[18]
Bruker microflex LT	16S rRNA and *secA1* genes	≥ 2.0/< 2.0 and ≥ 1.7	74/14	BioTyper ver. 3.1	14.9%/52.7%	ND	ND	[19]
Andromas	16S rRNA, *hsp65* genes, conventional	See note^b^	51/12	See note^b^	80.4%/17.6%	ND	ND	[21]
Bruker microflex LT	16S rRNA gene and conventional	≥ 2.0/< 2.0 and ≥ 1.7	43/9	BioTyper ver. 3.0.2	23.3%/20.9%	110/17	79.1%/9.3%	[20]

Abbreviations: ID, identification; Ni/Ns, number of isolates/number of species; DB, database; MLSA, multilocus sequence analysis.

^a^ In the present study, all 25 *Nocardia* clinical isolates collected were involved for validation of the commercial Bruker Biotyper database. While for the "in-house" database, five of the 25 *Nocardia* isolates were used for databased development, and the rest 20 isolates were used for "in-house" database validation. See details in Methods section.

^b^ An Andromas database (database version not specified) was employed in the study by Farfour *et al*. Criteria for correct identification to species level: percentage of common peaks is ≥ 68% and more than 10% difference between the first two best-match species. Criteria for correct identification to genus level: percentage of common peaks is ≥ 68% yet less than 10% difference between the first two best-match species.

### A proposed algorithm for species identification of *Nocardia* in clinical laboratories

**Fig 3:**
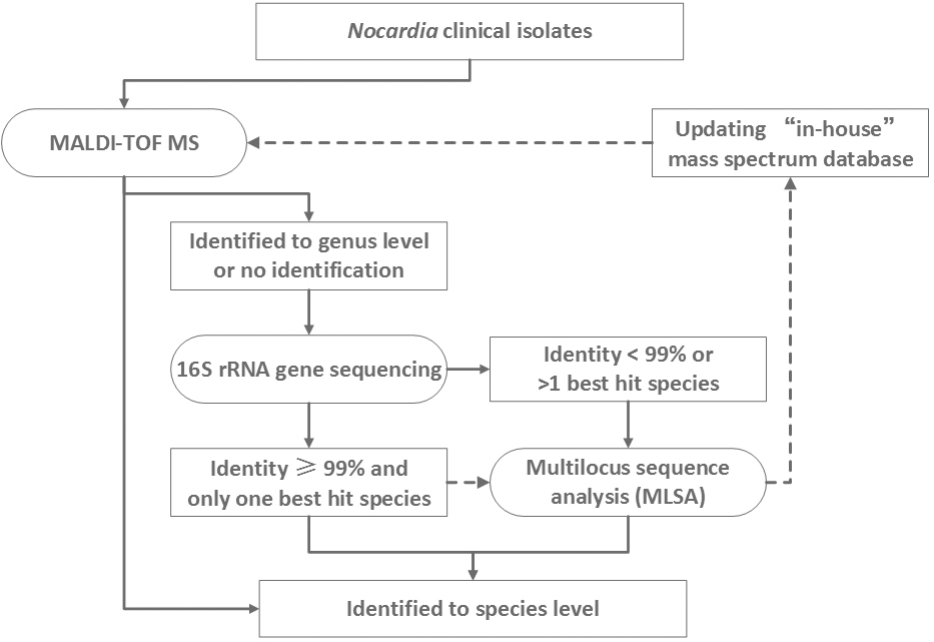
Proposed algorithm for laboratory identification of *Nocardia* isolates (solid lines) and establishment and expansion of ongoing “in-house” mass spectrum database (dashed lines).

Although the MLSA scheme was definitively able to assign species, identify novel species, and to differentiate within species, it has the disadvantage of relatively high costs, and requiring staff time for analysis of results. We propose an algorithm for species identification of *Nocardia* in the clinical laboratory (Fig 3), recommending that identification by MALDI-TOF MS using a database complemented by “in-house” protein profiles be attempted in the initial instance. For those isolates that cannot be identified to species level by MALDI-TOF MS, the 5’-end 606 bp partial 16S rRNA sequencing may be attempted. For any isolates with < 99% gene sequence similarity to a known archived strain sequence or those with two or more “best hit” species identities, MLSA could be undertaken for a definitive identification.

## Discussion

To date, more than 50 species of pathogenic *Nocardia* have been identified [31]. Since there are differences in prevalence of species between geographic regions, knowledge of species distribution is important. In the present study, *N*. *cyriacigeorgica* and *N*. *farcinica* were the most commonly encountered species, similar to that in Canada [16], but in contrast to a report from Taiwan where *Nocardia brasiliensis* was the most common [32]. In comparison, *Nocardia asteroides* was reported to be predominant in Switzerland [31], while *N*. *nova* was predominant in Australia [7].

Most studies examining identification of *Nocardia* have indicated that molecular techniques are required to achieve species assignment [6, 16, 31, 32]. Although polymorphisms in the 5’-end partial 16S rRNA gene is widely used to discriminate between *Nocardia* species, misidentification may occur due to high sequence similarity between certain species [6, 7, 16, 33], as exemplified by inability to distinguish *N*. *wallacei* from *N*. *transvalensis* in the present study, or due to multiple different copies of this gene, such as that in *N*. *nova* [9]. *N*. *wallacei* is very closely related to *N*. *transvalensis* and indeed was previously classified within the *N*. *transvalensis* complex. Using a more discriminatory method, in this case, a 5-locus MLSA, *N*. *wallacei* was assigned as an individual species, which supported findings by Conville *et al*. [12].

In this context then, MLSA has been carried out as an alternative technique for the identification of *Nocardia*. The most commonly used genes have been the 16S rRNA, *secA1*, *gyrB*, *rpoB* and *hsp65* genes. Different studies may employ different combination of the genes [11, 16, 34], yet it is notable that no single gene locus sequencing is sufficient to resolve a substantial number of *Nocardia* species [16]. In addition, the MLSA scheme can be used for examining intra-species genetic diversity [10, 11, 13]. Using this approach, Carrasco *et al*. has reported that species *N*. *nova* had highest intra-species heterogeneity, followed by *N*. *cyriacigeorgica* and *N*. *abscessus*, while *N*. *farcinica* was more conserved [11]. Although the diversity of *N*. *abscessus* and *N*. *nova* were not compared in this study due to only a limited number of isolates studied, we similarly found genetic heterogeneity within *N*. *cyriacigeorgica* where three subgroups were identified. Of note, these subgroups correspond to three *hsp65* genotypes proposed in previous studies [28, 29]. In the diagnostic algorithm proposed (Fig 3) for laboratory identification of *Nocardia*, MLSA has good clinical utility in definitive confirmative of species.

Application of sequencing-based methods in the routine work of clinical laboratories is restricted by high costs and the need for on-site sequencing facilities which potentially affect turn-around times. The recent introduction of MALDI-TOF MS platforms in clinical laboratories has revolutionized diagnostic microbiology [35]. However, its application in identification of *Nocardia* species required extra protein-extraction pretreatment of isolates [17, 30], and more importantly, may be limited by insufficient archiving of reference spectra within commercial MALDI-TOF MS databases [17–20]. In this study, the MSP analysis indicated significant differences between spectra of the clinical isolates studied and reference spectra of corresponding species contained in the Biotyper version 3.1 database (Bruker Daltonics), which yielded suboptimal results in species identification when used as the only database. However, the establishment of even a small “in-house” database significantly improved the identification capacity of the MALDI-TOF MS system, as also found previously [18, 20, 22, 23]. To improve the identification capacity of MALDI-TOF MS, it is important for MS databases to contain more reference mass spectra from type strains of different bacteria species, and also spectra representing different strains of the same species [35], as strains of the same species may have divergent mass spectra e.g. the case of *N*. *abscessus* clinical strain PUNC002 and PUNC017 found in the present study. With an ongoing expanded “in-house” database representing species that are the more frequently encountered, the utility and practicality of MALDI-TOF MS for *Nocardia* identification will be improved. However, before expanding any “in-house” mass spectrum database, we recommend that *Nocardia* isolates be analyzed by MLSA to ensure the high-quality of the “in-house” database (Fig 3). Moreover, comparing to MLSA, the MALDI-TOF MS system was unable to cluster *N*. *cyriacigeorgica* into subgroups. In this regard, MLSA is superior to MALDI-TOF MS for subgrouping *N*. *cyriacigeorgica*. Further studies addressing differences in MS profiles between *N*. *cyriacigeorgica* strains are warranted.

The major limitation of the current study is that relatively few isolates representing a small number of species was examined, and all isolates were from a single center and hence may not be generalizable across centers in China. Continuous expansion of the MALDI-TOF MS databases to include more species is necessary.

## Conclusions

In conclusion, we have evaluated MLSA scheme and MALDI-TOF MS for identification of *Nocardia* clinical isolates. MLSA showed superior discriminatory power compared with the 5’-end 606bp partial 16S rRNA gene sequencing for species identification of *Nocardia*. MALDI-TOF MS has good utility in rapidly and accurately providing species identification but is contingent on building up a robust library of reference spectra.

## Supporting Information

S1 Table Species of *Nocardia* and GenBank accession numbers of five gene sequences for six type strains of *Nocardia* studied. (DOCX)

S2 Table Haplotype nomenclature based on sequencing of five *Nocardia* genes for 25 clinical *Nocardia* isolates. (DOCX)
