# Supplementary material for: Accurate Identification of Common Pathogenic Nocardia Species: Evaluation of a Multilocus Sequence Analysis Platform and Matrix-Assisted Laser Desorption Ionization-Time of Flight Mass Spectrometry
Source: PLoS One. 2016 Jan 25;11(1):e0147487. doi: 10.1371/journal.pone.0147487 (PMC4726625; doi:10.1371/journal.pone.0147487)
Supplement: S1 Table — (DOCX) [file pone.0147487.s001.docx]

**S1 Table. Species of *Nocardia* and GenBank accession numbers of five gene sequences for six type strains of *Nocardia* studied.**

| **Strain** | **Species** | **GenBank accession no. of gene sequences** | | | | |
| --- | --- | --- | --- | --- | --- | --- |
|  |  | ***gyrB* (482 bp)** | **16S rRNA (462 bp)** | ***secA1* (445 bp)** | ***hsp65* (401 bp)** | ***rpoB* (400 bp)** |
| DSM 44432^T^/  ATCC 23824^T^ | *N. abscessus* | JN041252 | JN041489 | JN041963 | JN041726 | JN215593 |
| DSM 44491^T^  /ATCC BAA-280^T^ | *N. africana* | JN041368 | JN041605 | JN042079 | JN041842 | JN215709 |
| DSM 44731^T^ | *N. arthritidis* | JN041235 | JN041472 | JN041946 | JN041709 | JN215576 |
| ATCC 19247^T^ | *N. asteroids* | JN041222 | JN041459 | JN041933 | JN041696 | JN215563 |
| JCM 10666^T^ | *N. beijingensis* | JN041231 | JN041468 | JN041942 | JN041705 | JN215572 |
| ATCC 19296^T^ | *N. brasiliensis* | JN041298 | JN041535 | JN042009 | JN041772 | JN215639 |
| DSM 43397^T^  /ATCC 6847^T^ | *N. carnea* | JN041362 | JN041599 | JN042073 | JN041836 | JN215703 |
| DSM 44484^T^ | *N. cyriacigeorgica* | JN041323 | JN041560 | JN042034 | JN041797 | JN215664 |
| NRRL B-2089^T^  /ATCC 3318^T^ | *N. farcinica* | JN041445 | JN041682 | JN042156 | JN041919 | JN215786 |
| DSM 44732^T^ | *N. higoensis* | AB450789 | GQ376169 | EU178747 | DQ789013 | DQ085142 |
| DSM 44496^T^ | *N. ignorata* | JN041217 | JN041454 | JN041928 | JN041691 | JN215558 |
| DSM 44670^T^ | *N. niigatensis* | AB427101 | GQ853079 | DQ360278 | AY903629 | DQ085137 |
| DSM 43242^T^  /ATCC 14629^T^ | *N. otitidiscaviarum* | JN041275 | JN041512 | JN041986 | JN041749 | JN215616 |
| DSM 44386^T^  /ATCC BAA-278^T^ | *N. paucivorans* | JN041350 | JN041587 | JN042061 | JN041824 | JN215691 |
| DSM 44730^T^ | *N. pneumoniae* | JN041228 | JN041465 | JN041939 | JN041702 | JN215569 |
| DSM 44599^T^ | *N. puris* | GQ496095 | GQ217500 | EU178750 | AY903632 | DQ085140 |
| NRRL B-16037^T^  /ATCC 6865^T^ | *N. transvalensis* | JN041286 | JN041523 | JN041997 | JN041760 | JN215627 |
| DSM 44445^T^ | *N. veteran* | JN041365 | JN041602 | JN042076 | JN041839 | JN215706 |
| JCM 10988^T^ | *N. vinacea* | JN041293 | JN041530 | JN042004 | JN041767 | JN215634 |
| DSM 45136^T^  /ATCC 49873^T^ | *N. wallacei* | JN041290 | JN041527 | JN042001 | JN041764 | JN215631 |
| ATCC 25592^T^ | *Gordonia bronchialis* | CP001802 | CP001802 | CP001802 | CP001802 | CP001802 |

Abbreviations: DSM, German Collection of Microorganisms and Cell Cultures; NRRL, United States Department of Agriculture; ^T^: Type strains.
