# Supplementary material for: Accurate Identification of Common Pathogenic Nocardia Species: Evaluation of a Multilocus Sequence Analysis Platform and Matrix-Assisted Laser Desorption Ionization-Time of Flight Mass Spectrometry
Source: PLoS One. 2016 Jan 25;11(1):e0147487. doi: 10.1371/journal.pone.0147487 (PMC4726625; doi:10.1371/journal.pone.0147487)
Supplement: S2 Table — (DOCX) [file pone.0147487.s002.docx]

**S2 Table. Haplotype nomenclature based on sequencing of five *Nocardia* genes for 25 clinical *Nocardia* isolates.**

| **Species identification by MLSA** | **Strain**  **ID no.** | ***gyrB* (482 bp)** | | **16S rRNA (462 bp)** | | ***secA1* (445 bp)** | | ***hsp65* (401 bp)** | | ***rpoB* (400 bp)** | |
| --- | --- | --- | --- | --- | --- | --- | --- | --- | --- | --- | --- |
|  |  | **Haplotype** | **Accession no.** | **Haplotype** | **Accession no.** | **Haplotype** | **Accession no.** | **Haplotype** | **Accession no.** | **Haplotype** | **Accession no.** |
| *N. abscessus* | PUNC002 | 5 | KU052086 | 1 | KT985912 | 9 | KU052161 | 8 | KU052111 | 9 | KU052136 |
| *N. abscessus* | PUNC017 | 6 | KU052101 | 1 | KT985927 | 9 | KU052176 | 9 | KU052126 | 9 | KU052151 |
| *N. cyriacigeorgica* | PUNC003 | 16 | KU052087 | 2 | KT985913 | 4 | KU052162 | 5 | KU052112 | 3 | KU052137 |
| *N. cyriacigeorgica* | PUNC005 | 16 | KU052089 | 2 | KT985915 | 5 | KU052164 | 5 | KU052114 | 4 | KU052139 |
| *N. cyriacigeorgica* | PUNC006 | 12 | KU052090 | 2 | KT985916 | 2 | KU052165 | 1 | KU052115 | 7 | KU052140 |
| *N. cyriacigeorgica* | PUNC007 | 14 | KU052091 | 2 | KT985917 | 1 | KU052166 | 1 | KU052116 | 5 | KU052141 |
| *N. cyriacigeorgica* | PUNC010 | 9 | KU052094 | 2 | KT985920 | 4 | KU052169 | 3 | KU052119 | 1 | KU052144 |
| *N. cyriacigeorgica* | PUNC011 | 9 | KU052095 | 2 | KT985921 | 4 | KU052170 | 3 | KU052120 | 1 | KU052145 |
| *N. cyriacigeorgica* | PUNC013 | 9 | KU052097 | 2 | KT985923 | 4 | KU052172 | 3 | KU052122 | 1 | KU052147 |
| *N. cyriacigeorgica* | PUNC016 | 10 | KU052100 | 2 | KT985926 | 1 | KU052175 | 2 | KU052125 | 5 | KU052150 |
| *N. cyriacigeorgica* | PUNC018 | 12 | KU052102 | 2 | KT985928 | 3 | KU052177 | 4 | KU052127 | 8 | KU052152 |
| *N. cyriacigeorgica* | PUNC019 | 13 | KU052103 | 2 | KT985929 | 1 | KU052178 | 1 | KU052128 | 6 | KU052153 |
| *N. cyriacigeorgica* | PUNC021 | 11 | KU052105 | 2 | KT985931 | 6 | KU052180 | 3 | KU052130 | 2 | KU052155 |
| *N. cyriacigeorgica* | PUNC025 | 15 | KU052109 | 2 | KT985935 | 3 | KU052184 | 1 | KU052134 | 8 | KU052159 |
| *N. farcinica* | PUNC001 | 1 | KU052085 | 3 | KT985911 | 10 | KU052160 | 6 | KU052110 | 10 | KU052135 |
| *N. farcinica* | PUNC004 | 2 | KU052088 | 3 | KT985914 | 13 | KU052163 | 6 | KU052113 | 12 | KU052138 |
| *N. farcinica* | PUNC008 | 1 | KU052092 | 3 | KT985918 | 14 | KU052167 | 6 | KU052117 | 10 | KU052142 |
| *N. farcinica* | PUNC009 | 4 | KU052093 | 3 | KT985919 | 11 | KU052168 | 6 | KU052118 | 11 | KU052143 |
| *N. farcinica* | PUNC012 | 1 | KU052096 | 3 | KT985922 | 10 | KU052171 | 6 | KU052121 | 10 | KU052146 |
| *N. farcinica* | PUNC014 | 3 | KU052098 | 3 | KT985924 | 11 | KU052173 | 6 | KU052123 | 12 | KU052148 |
| *N. farcinica* | PUNC015 | 2 | KU052099 | 3 | KT985925 | 12 | KU052174 | 6 | KU052124 | 14 | KU052149 |
| *N. farcinica* | PUNC022 | 1 | KU052106 | 3 | KT985932 | 14 | KU052181 | 6 | KU052131 | 13 | KU052156 |
| *N. farcinica* | PUNC023 | 4 | KU052107 | 3 | KT985933 | 12 | KU052182 | 6 | KU052132 | 14 | KU052157 |
| *N. otitidiscaviarum* | PUNC024 | 7 | KU052108 | 4 | KT985934 | 8 | KU052183 | 7 | KU052133 | 16 | KU052158 |
| *N. wallacei* | PUNC020 | 8 | KU052104 | 5 | KT985930 | 7 | KU052179 | 10 | KU052129 | 15 | KU052154 |
